# Crosstalk Between Histone and DNA Methylation in Regulation of Retinal Matrix Metalloproteinase-9 in Diabetes

**DOI:** 10.1167/iovs.17-22706

**Published:** 2017-12

**Authors:** Arul J. Duraisamy, Manish Mishra, Renu A. Kowluru

**Affiliations:** Kresge Eye Institute, Wayne State University, Detroit, Michigan, United States

**Keywords:** diabetic retinopathy, DNA methylation, epigenetics, histone methylation, matrix metalloproteinase-9

## Abstract

**Purpose:**

Diabetes activates matrix metalloproteinase-9 (MMP-9), and MMP-9 via damaging retinal mitochondria, activates capillary cell apoptosis. *MMP-9* promoter has binding sites for many transcription factors, and in diabetes its promoter undergoes epigenetic modifications, including histone modifications and DNA methylation. Enhancer of Zeste homolog 2 (Ezh2), which catalyzes dimethylation/trimethylation of histone 3 lysine 27 (H3K27me2 and me3), is also associated with DNA methylation. Our aim was to investigate link(s) between histone and DNA modifications in the regulation of *MMP-9*.

**Methods:**

Using human retinal endothelial cells, and also retinal microvessels from diabetic rats, effect of hyperglycemia on H3K27me3, and recruitment of Ezh2 at the *MMP-9* promoter were quantified by chromatin-immunoprecipitation technique. Role of H3K27 trimethylation in regulating DNA methylation-transcription of *MMP-9* was determined by regulating Ezh2 by its specific siRNA and also a pharmacologic inhibitor.

**Results:**

Hyperglycemia elevated H3K27me3 levels and the recruitment of Ezh2 at the *MMP-9* promoter, and increased the enzyme activity of Ezh2. Inhibition of Ezh2 attenuated recruitment of both DNA methylating (Dnmt1) and hydroxymethylating (Tet2) enzymes and 5 hydroxymethyl cytosine at the same region of the *MMP-9* promoter, and prevented increase in *MMP-9* transcription and mitochondrial damage.

**Conclusions:**

Activation of Ezh2 in diabetes, via trimethylation of H3K27, facilitates recruitment of the enzymes responsible for regulation of DNA methylation of the *MMP-9* promoter, resulting in its transcriptional activation. Thus, a close crosstalk between H3K27 trimethylation and DNA methylation in diabetes plays a critical role in the maintenance of cellular epigenetic integrity of *MMP-9*.

Diabetic retinopathy is the leading cause of vision loss in young adults, and despite ongoing research in the field, its etiology remains elusive. Animal models have clearly documented that apoptosis of retinal cells, including vascular and nonvascular cells, proceeds the development of histopathology characteristic of diabetic retinopathy,^1–3^ and mitochondrial damage is implicated in the accelerated apoptosis of capillary cells.^4,5^ Our previous work has shown that diabetes activates gelatin matrix metalloproteinases (MMP-2 and MMP-9) in the retina and its capillary cells, and this activation is an early event in the pathogenesis of diabetic retinopathy. Accumulation of activated MMPs in the mitochondria damage their membranes, leading to transport of cytochrome C into the cytosol, and apoptosis of capillary cells.^6–8^

The promoter of *MMP-9* has many transcriptional factor binding sites,^9^ and the binding of transcriptional factors AP-1 and nuclear factor-*k*B (NF-*k*B) is increased at the retinal *MMP-9* promoter in diabetes.^10,11^ In addition to transcription factors, epigenetic modifications including DNA methylation and histone modifications, can also regulate the gene expression without altering the DNA sequence, and the same gene can be regulated by multiple epigenetic modifications.^12,13^ We have shown that in diabetes, lysine 9 of histone 3 (H3K9) of retinal *MMP-9* promoter is hyperacetylated, facilitating the binding of NF-*k*B.^11^ Histone 3, however, can undergo at least 17 different posttranslational modifications, and methylation is one of the most abundant modifications, which is mainly considered as a gene repressive mark.^14^ Trimethylation of lysine 27 of histone 3 (H3K27me3) is a relatively stable inheritable repressive histone mark and enhancer of Zeste homolog 2 (Ezh2), the catalytic component of the polycomb repressive complex 2 (PRC2) histone methyltransferase, catalyzes dimethylation (me2) and trimethylation (me3) of H3K27, repressing the expression of many target genes including *MMP-9*.^15,16^ Ezh2 expression is increased in retinal endothelial cells in diabetes,^17^ but, its role in regulating *MMP-9* expression remains unclear. Ezh2 is also directly involved in DNA methylation,^18,19^ and in diabetes *MMP-9* promoter undergoes dynamic DNA methylation. Despite increased recruitment of DNA methyltransferase 1 (Dnmt1) at the *MMP-9* promoter in diabetes, our results have shown that 5 methyl cytosine (5mC) levels are decreased. The reason for the decrease in 5mC appears to be the concomitant increase in the binding of hydroxymethylase, ten-eleven translocase 2 (Tet2), at the same site of the promoter, and increase in 5 hydroxymethyl cytosine (5hmC), in turn, activates *MMP-9* transcription.^20^ However, the crosstalk between histone methylation and DNA methylation in the regulation of retinal *MMP-9* in diabetes remains to be investigated.

This study aims to investigate link(s) between histone and DNA modifications in the regulation of *MMP-9* expression in diabetes. Using both in vitro (human retinal endothelial cells; HRECs) and in vivo (retinal microvessels from diabetic rats) models of diabetic retinopathy, and retinal microvessels from human donors with documented diabetic retinopathy, we have investigated the effect of hyperglycemia on H3K27me3 and Ezh2 recruitment at the AP-1 region of the *MMP-9* promoter. A crosstalk between H3K27 methylation and DNA methylation of *MMP-9* promoter is investigated by pharmacologic and molecular regulation of Ezh2.

## Methods

HRECs, purchased from Cell Systems Corporation (Kirkland, WA, USA), were cultured in Dulbecco's modified Eagle medium (DMEM)-F12 containing 10% heat-inactivated fetal bovine serum, endothelial cell growth supplement (15 μg/mL), insulin transferrin selenium (1%), Glutamax (1%), and antibiotic/antimycotic (1%) in an environment of 95% O_2_ and 5% CO_2,_ as described previously.^21^ Cells from the fifth to seventh passage were incubated in 5- or 20-mM glucose for 4 days in the presence or absence of Ezh2 inhibitor, 3-Deazaneplanocin A (5-μM DZNep; EMD Millipore, Billerica, MA, USA), in a medium containing instead 1% fetal bovine serum, 9% Nu-serum, and 2- to 5-μg/mL endothelial cell growth supplement. Parallel osmotic control included HRECs incubated in 20-mM mannitol.

A batch of cells were transfected with *Ezh2*-siRNA (Santa Cruz Biotechnology, Santa Cruz, CA, USA) using the procedures described previously,^5,20^ followed by incubation in 5- or 20-mM glucose media for four days. The parallel controls included cells scrambled RNA transfected cells incubated in 5- or 20-mM glucose.

Wistar rats (∼200g; 7–8 weeks), obtained from Harlan Labs (South Easton, MA, USA) were made diabetic by intraperitoneal injection of streptozotocin (55-mg/kg body weight). After 2 months of diabetes, the rats were killed by CO_2_ asphyxiation and the retina was collected immediately.^20^ Age-matched normal rats served as their controls. The treatment of the animals was in accordance with the guidelines of the ARVO Resolution on the Use of Animals in Research.

Human donor retinas, isolated from eye globes obtained from donors between 34 and 76 years of age (supplied by the Eversight, Ann Arbor, MI, USA) with 10 to 43 years of diabetes and documented retinopathy, and their age-matched nondiabetic donors (Table 1), were used to isolate microvessels.^22,23^

**Table 1:**
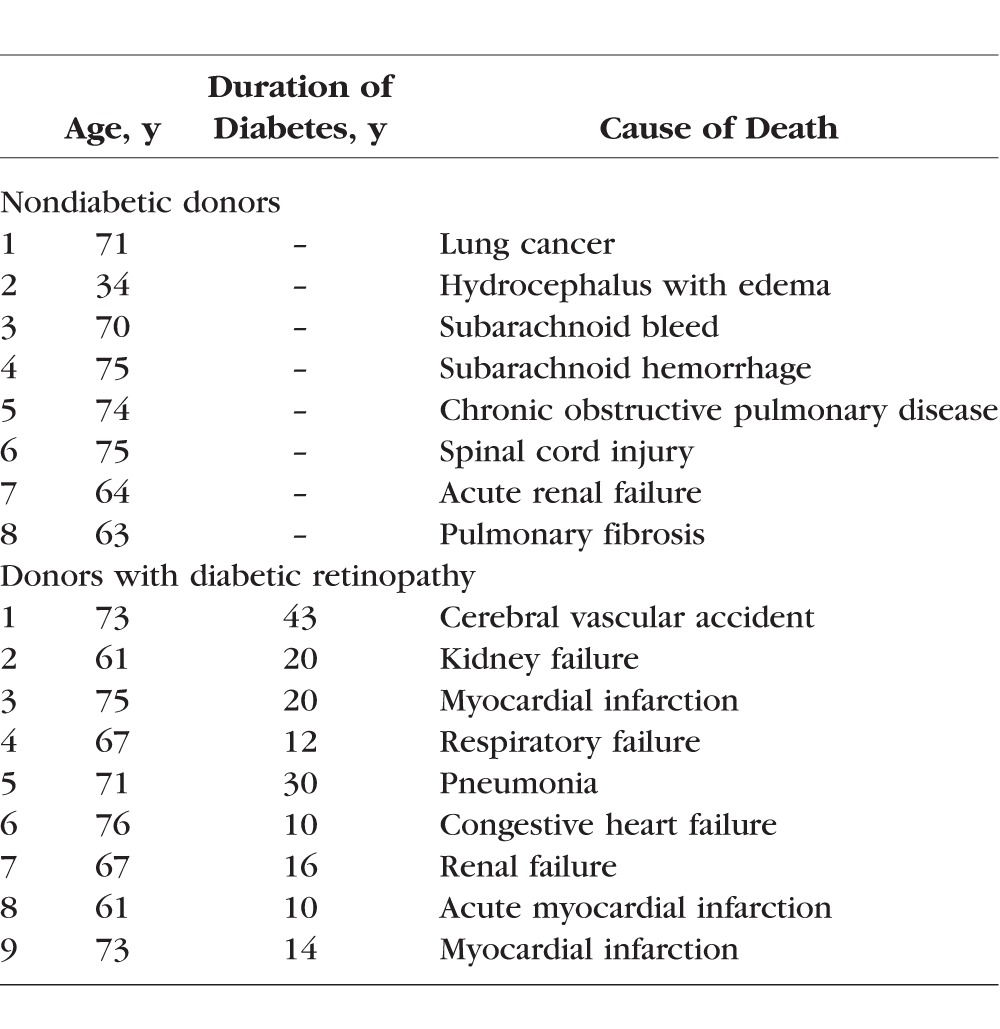
Human Donors

Retinal microvessels were prepared by osmotic shock method by incubating the retina (rats/human) in distilled water for 1 hour at 37°C with gentle shaking. The microvessels were isolated under microscope with repeated inspiration and ejection using Pasteur pipette. As reported previously, these microvessel preparations are largely devoid of nonvascular components.^22,23^ The microvessels rinsed with sterile PBS were either crosslinked with 1% paraformaldehyde for chromatin immunoprecipitation (ChIP), or used for RNA isolation with Trizol reagent.

ChIP was performed in the cross-linked microvessels, sonicated in ChIP lysis buffer. Protein-DNA complex (100 μg) was immunoprecipitated with either H3K27me3 or Ezh2 or Dnmt1 or Tet2 antibody (ab6002, ab191080, ab13537, and ab135087, respectively; Abcam, Cambridge, MA, USA). IgG (ab171870) was used as an antibody control. The antibody chromatin complex was precipitated using Protein A Agarose/Salmon Sperm DNA (EMD Millipore), washed and de-crosslinked at 65°C for 6 hours followed by DNA isolation with phenol:choloroform:isoamylalcohol using the methods reported previously.^5,11^ The DNA was resuspended in water and relative abundance of methylated H3K27 and enzyme binding at *MMP-9* promoter was quantified by real-time quantitative (q)PCR using primers specific for *MMP-9* promoter proximal AP-1 binding site (Table 2). The target values were normalized to input controls, respectively, to obtain fold change. The specificity of the assay was validated by resolving the PCR products in 2% agarose gel.^10,11^

**Table 2:**
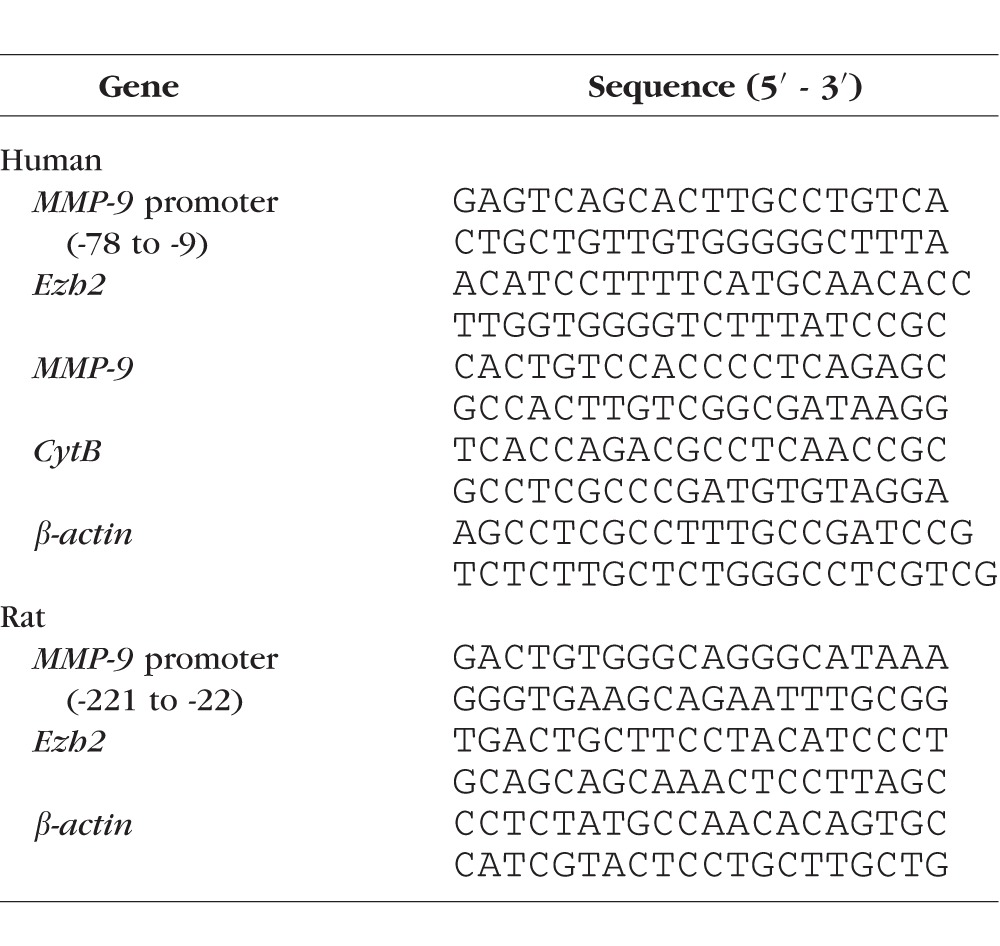
Primer Sequences

Histone methyltransferase, Ezh2, activity was measured in nuclear fraction prepared following the manufacturer's instruction (EPIGENTEK, Farmingdale, NY, USA). Briefly, nuclear fraction (10 μg) was incubated in H3 substrate coated microplate with a methyl donor for 1 hour, and the formation of methylated H3K27 was detected using specific antibody. The activity was represented as percentage controls.

Levels of 5hmC were quantified in sonicated DNA, which was immunoprecipitated for 5hmC using hydroxymethylated DNA Immunoprecipitation (hMeDIP) Kit (EPIGENTEK). The enriched 5hmC at the *MMP-9* promoter was analyzed by qPCR using specific primers.^10,20^

Immunofluorescence technique was performed to confirm the effect of Ezh2 inhibition on MMP-9. HRECs grown on coverslips, exposed to 5- or 20-mM glucose, in the presence or absence of Ezh2 inhibitor DZNep, were fixed with paraformaldehyde and incubated with antibodies against MMP-9 and cytochrome oxidase IV (CoxIV, a mitochondrial marker). Secondary antibodies included a DyLight 488-labelled for MMP-9 and Texas red-conjugated for CoxIV mounting. The cells were washed and mounted with DAPI containing medium and the images were visualized using Zeiss ApoTome fluorescence microscope at ×40 magnification (Carles Zeiss, Inc., Chicago, IL, USA).

Gene expression of *Ezh2*, *MMP-9*, and cytochrome B (*CytB*) were quantified using specific primers using real time qPCR (Table 2). The specific products were confirmed by SYBR green single melt curve analysis. The results were normalized to the expression of the housekeeping gene *β-actin*, and relative fold change was calculated using delta delta Ct method.^11,24^

MMP-9 activity was quantified by fluorescence kit (SensoLyte Plus 520 MMP-9 Assay Kit; AnaSpec, Inc., Fremont, CA, USA) using approximately 30-μg protein. Cleavage of the fluorogenic peptide, induced by MMP-9, was measured at 490-nm excitation and 520-nm emission wavelengths.^25^

Cell apoptosis was determined in 20 μg of the cytoplasmic protein by the Cell Death Detection ELISAPLUS kit from Roche Diagnostics (Indianapolis, IN, USA) using monoclonal antibodies against DNA and histones, and peroxidase conjugated anti-DNA and biotin-labeled antihistone.^8^

### Statistical Analysis

Data are presented as mean ± SD. Comparison between groups were made using one-way ANOVA followed by Dunn's *t*-test and a *P* value less than 0.05 was considered significant.

## Results

**Figure 1:**
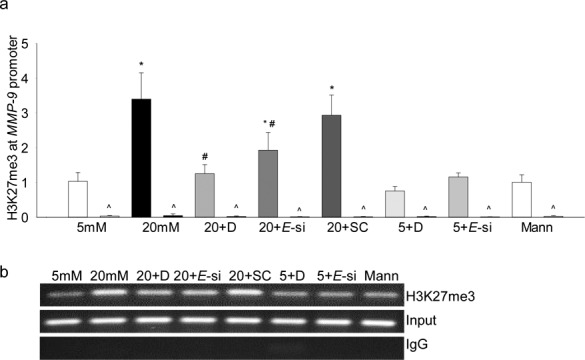
Effect of high glucose on trimethylation of H3K27 and its regulation by Ezh2. HRECs incubated in high glucose for 4 days in the presence or absence of DZNep (5 μM), or transfected with Ezh2-siRNA, were analyzed for (a) H3K27me3 levels at the MMP-9 promoter by immunoprecipitating H3K27me3 in the cross-linked samples, followed by PCR using the primers for the AP-1 binding region of the MMP-9 promoter. IgG (^) was used as an antibody control. Ct values were normalized with the values from input by delta delta Ct method. (b) Agarose gel picture showing the band intensity. Values are represented as mean ± SD from three different cell preparations; 5 and 20 mM = 5- and 20-mM glucose, respectively; 5 + D and 20 + D = cells in 5- and 20-mM glucose, respectively, with DZNep; 20+E-si and 20+SC = Ezh2-siRNA or scrambled RNA cells in 20-mM glucose; 5 + E-si = Ezh2-siRNA transfected cells in 5-mM glucose; Mann = 20-mM mannitol. *P < 0.05 compared with 5-mM glucose, #P < 0.05 compared with 20-mM glucose.

Histone modification is a complex process and many repressive and activating modifications can regulate the expression of a gene,^26^ the effect of high glucose on methylation of H3K27 at the *MMP-9* promoter was investigated by ChIP technique. High glucose increased methylation of H3K27, the levels of H3K27me3 were increased by approximately 4-fold in the AP-1 region of the *MMP-9* promoter. At the same site, IgG control values were less than 1% of the values obtained from cell precipitated with H3K27me3 antibodies (Fig. 1a), accompanying Figure 1b shows the band intensity on a 2% agarose gel. However, addition of 20-mM mannitol, instead of 20-mM glucose, had no effect on H3K27 methylation.

**Figure 2:**
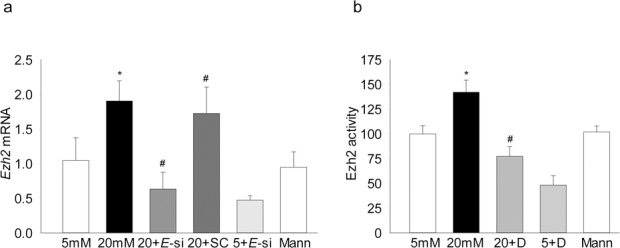
Effect of high glucose on Ezh2: (a) Ezh2 mRNA levels were quantified in the cDNA by real time PCR using β-actin as the housekeeping gene. (b) The enzyme activity of Ezh2 was determined using Ezh2 activity/inhibition assay kit, and the values obtained from cells in 5-mM glucose are considered as 100%. Values are mean ± SD of three to four experiments, each done in duplicate. *P < 0.05 compared with 5-mM glucose, #P < 0.05 compared with 20-mM glucose.

Because Ezh2 specifically methylates H3K27,^16^ the effect of high glucose on Ezh2 was investigated. As shown in Figure 2a, its mRNA levels were elevated by approximately 2-fold in HRECs incubated in high glucose compared with cells in normal glucose. Increase in Ezh2 expression was accompanied by approximately 45% increase in its enzyme activity (Fig. 2b).

**Figure 3:**
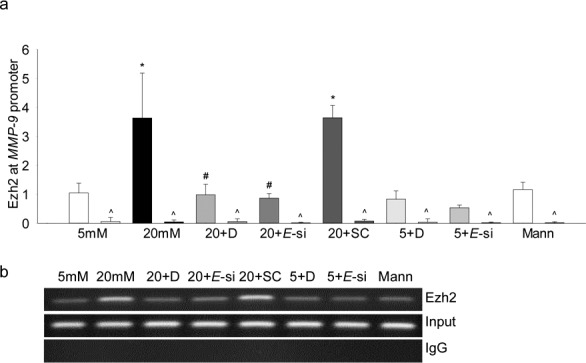
Effect of high glucose on Ezh2 recruitment at the MMP-9 promoter. The binding of Ezh2 at the MMP-9 promoter was quantified using Ezh2 monoclonal antibody, (a) followed by amplification of the promoter region by real time qPCR. (b) Product sizes were confirmed on 2% agarose gel. *P < 0.05 compared with 5-mM glucose; #P < 0.05 compared with 20-mM glucose.

**Figure 4:**
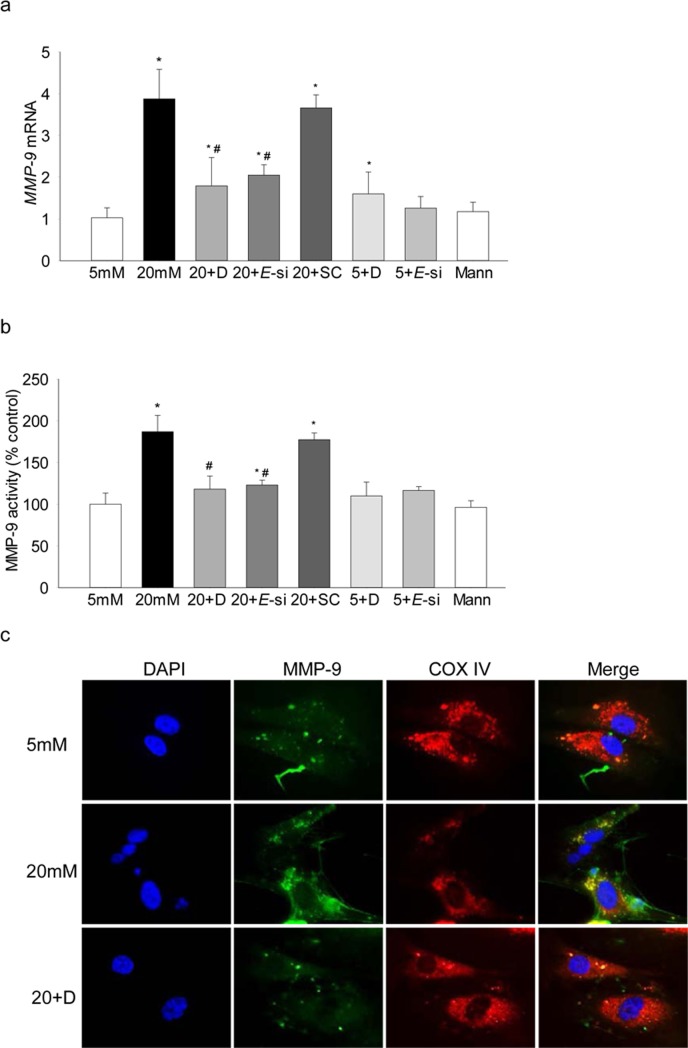
Effect of regulation of Ezh2 on MMP-9: (a) mRNA levels of MMP-9 were measured using real time qPCR, and each measurement was made in duplicate in three to five samples in each group. (b) Enzyme activity of MMP-9 was quantified in by an ELISA method using a fluorescence kit. (c) Mitochondrial localization of MMP-9 was determined by immunofluorescence using DyLight 488-conjugated secondary antibody for MMP-9 and Texas red-conjugated for CoxIV. *P < 0.05 compared with 5-mM glucose; #P < 0.05 compared with 20-mM glucose.

To investigate the role of Ezh2 in regulation of histone methylation, its binding at the *MMP-9* promoter was determined, and as shown in Figure 3, high glucose increased Ezh2 recruitment by over 4-fold. The role of Ezh2 in regulation of MMP-9 was confirmed using both pharmacologic (DZNep) and molecular (siRNA) inhibitors of Ezh2; addition of DZNep in high-glucose medium attenuated increase in H3K27me3 levels and Ezh2 recruitment at the *MMP-9* promoter (Figs. 1, 3), however, inclusion of DZNep in cells incubated in 5-mM glucose had no effect on H3K27me3 levels and Ezh2 binding. In the same cells, DZNep also ameliorated glucose-induced increase in *MMP-9* expression, the values in high glucose+DZNep cells were reduced by approximately 2-fold compared with the cells in high glucose alone, but they remained significantly higher compared with the cells in normal glucose. Consistent with this, although *MMP-9* expression in 5-mM glucose+DZNep and 20-mM glucose+DZNep were not different from each other, cells incubated in 5-mM glucose+DZNep had an approximately 1.8-fold increase in *MMP-9* compared with cells in 5-mM glucose alone (Fig. 4). However, transfection of cells with *Ezh2*-siRNA, but not with scrambled RNA, attenuated glucose-induced increase in H3K27me3 levels and Ezh2 recruitment at the *MMP-9* promoter (Figs. 1, 2), and ameliorated increase in both MMP-9 expression and activity (Figs. 4a, 4b). Consistent with our previous results showing increased mitochondrial levels of MMP-9 in hyperglycemia,^6^ as shown in Figure 4c, Ezh2 inhibition also ameliorated glucose-induced increase in mitochondrial accumulation of MMP-9.

**Figure 5:**
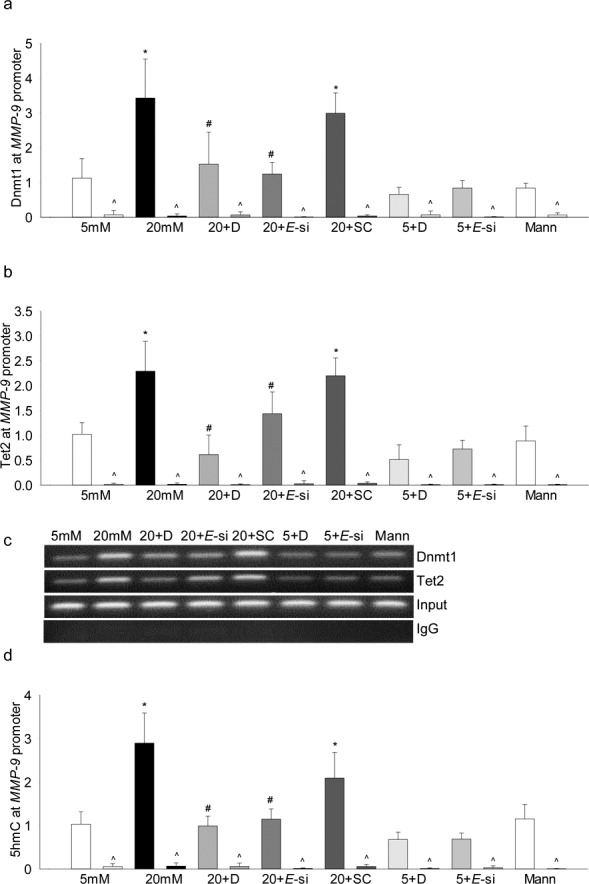
Ezh2 regulates DNA methylation of MMP-9 promoter. Recruitment of (a) Dnmt1 and (b) Tet2 at the MMP-9 promoter was measured in the cells incubated in high glucose ± DZNep, or transfected with Ezh2-siRNA or scrambled RNA (SC) by ChIP technique. (c) A representative agarose gel showing the accompanying band intensities. (d) The levels of 5hmC were quantified using hMeDIP immunoprecipitation kit. *P < 0.05 compared with 5-mM glucose; #P < 0.05 compared with 20-mM glucose.

Ezh2, in addition to methylating H3K27, can also regulate DNA methylation by allosterically binding with Dnmt1.^18,19^ Among the DNA methylating-hydroxymethylating family of enzymes, our previous work has shown increased mRNA levels of Dnmt1 and Tet2 in the retinal capillary cells in diabetes, and a dynamic DNA methylation-hydroxymethylation is implicated in the regulation of *MMP-9* transcription.^20^ Consistent with our previous results, despite increased Dnmt1 recruitment at the *MMP-9* promoter, 5hmC levels and Tet2 binding were increased by 2.5-4 fold, however, regulation of Ezh2 by DZNep or its siRNA attenuated glucose-induced increase in the recruitment of both Dnmt1 and Tet2 at the promoter, and also ameliorated increase in 5hmC levels (Figs. 5a–d), suggesting a crosstalk between H3K27me3 and dynamic DNA methylation.

**Figure 6:**
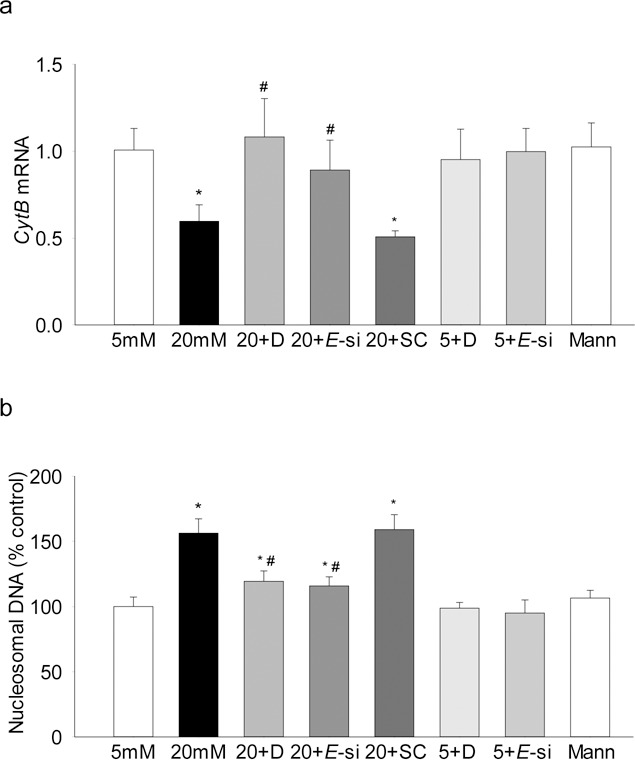
Regulation of Ezh2 activation prevents glucose-induced decrease in mtDNA transcription and cell apoptosis. (a) mRNA levels of mtDNA-encoded Cytb were quantified by real time qPCR using β-actin as the housekeeping gene. (b) Cell apoptosis was determined using an ELISA kit for histone-associated-DNA-fragments. The values are represented as mean ± SD from three to four samples/group, each measurement made in duplicate. *P < 0.05 compared with 5-mM glucose; #P < 0.05 compared with 20-mM glucose.

Because glucose-induced increase in MMP-9 is implicated in mitochondrial damage,^6,27^ the effect of regulation of Ezh2 on mtDNA transcription was investigated, and as shown in Figure 6a, glucose-induced decrease in *CytB* was ameliorated by inhibition of Ezh2 by DZNep or its siRNA. Consistent with the amelioration of mtDNA damage, Ezh2 inhibition also protected glucose-induced increase in capillary cell apoptosis (Fig. 6b). The values obtained from cells incubated in the presence of DZNep, or cells transfected with *Ezh2*-siRNA, and exposed to 20-mM glucose were not significantly different from those obtained from cells in 5-mM glucose.

**Figure 7:**
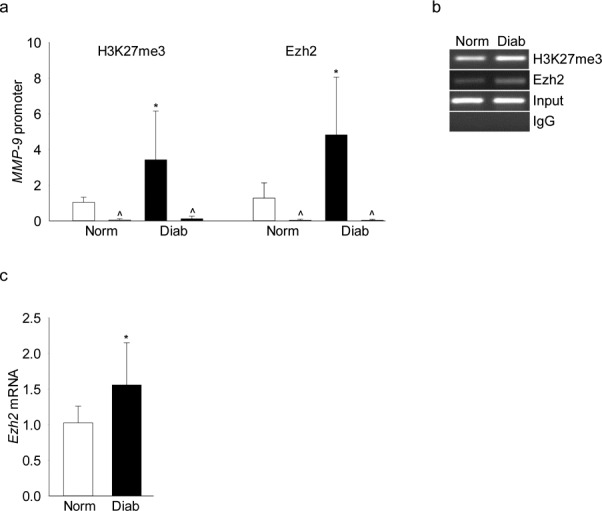
Diabetes increases H3K27me3 and Ezh2 recruitment at the MMP-9 promoter. Rat retinal microvessels were analyzed for (a) H3K27me3 and Ezh2 binding at the MMP-9 promoter by ChIP technique using IgG (^) as an antibody control. (b) Representative picture of an agarose gel showing the band intensities (c) Ezh2 mRNA levels were quantified in the cDNA by real time qPCR, and β-actin was used as a housekeeping gene. Values are represented as mean ± SD obtained from five to seven rats in each group. Norm and Diab = normal and diabetic rats, respectively. *P < 0.05 compared with normal.

To confirm the results in an in vivo model, retinal microvessels from rats diabetic for 2 months were analyzed. As with HRECs, diabetes increased H3K27me3 levels at the *MMP-9* promoter by approximately 4-fold, and this was accompanied by an increase in Ezh2 recruitment at the same site of the promoter (Fig. 7a); accompanying gel picture (Fig. 7b) represents the band intensity on a 2% agarose gel. Figure 7c shows significant increase in *Ezh2* mRNA in the same microvessel preparation.

**Figure 8:**
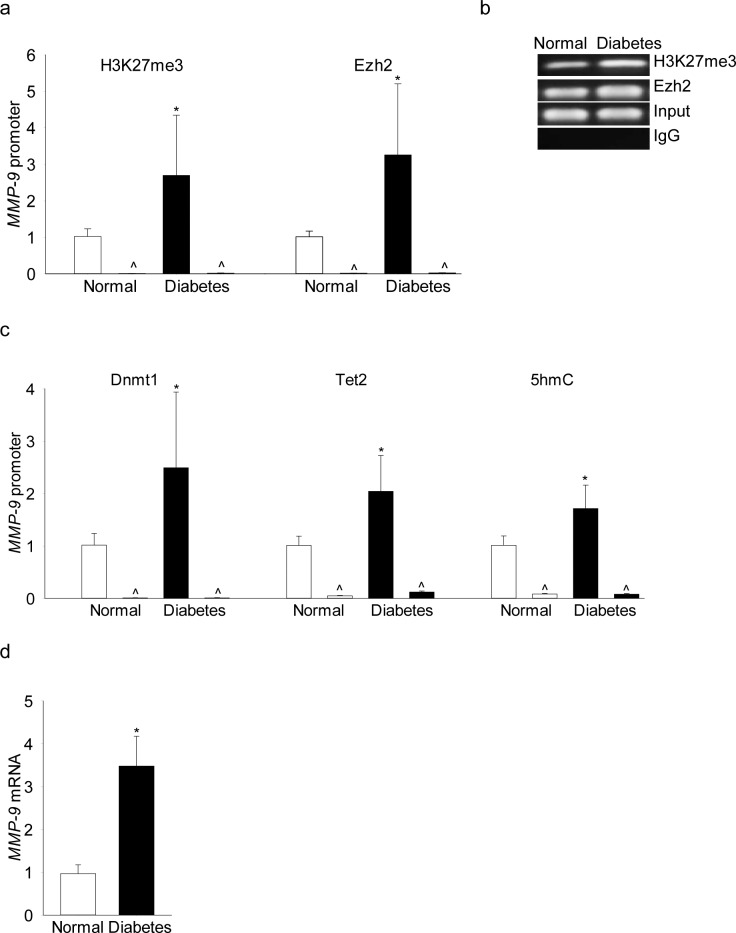
Retinal microvessels from human donors with diabetic retinopathy have increased H3K27me3 and Ezh2 recruitment at their MMP-9 promoter. (a) H3K27me3 and recruitment of (b) Ezh2 or (c) Dnmt1/Tet2 was quantified by ChIP technique using specific antibodies, and 5hmC by hMeDIP immunoprecipitation kit. (d) MMP-9 mRNA was quantified by real-time qPCR using gene-specific primers, in the retinal microvessels from human donors with 10 to 43 years of diabetes and documented retinopathy (diabetes), and their age-matched nondiabetic donors (normal). Amplification of the promoter region of MMP-9 was performed by qPCR, and the product sizes were confirmed on 2% agarose gel. Values are represented as mean ± SD; *P < 0.05 versus nondiabetic.

To transition to the human disease, retinal microvessels from human donors with documented diabetic retinopathy were analyzed. Consistent with the results from in vitro and in vivo models, H3K27me3 levels were elevated by over 2.5-fold and Ezh2 binding was increased by approximately 4-fold at the *MMP-9* promoter in microvessels from donors with diabetic retinopathy compared with their age-matched nondiabetic donors (Fig. 8a). Figure 8b is included to show the band intensity. In the same donors with documented diabetic retinopathy, recruitment of Dnmt1 and Tet2 and 5hmC levels were also significantly higher and *MMP-9* expression was elevated by approximately 4-fold (Figs. 8c, 8d).

## Discussion

In diabetes, activation of gelatinase MMPs (MMP-2 and MMP-9) in the retina is an early event, and activated MMPs, by damaging the mitochondria, activate the apoptotic machinery, culminating in the loss of capillary cells, a phenomenon seen before histopathology characteristic of diabetic retinopathy can be observed.^6,27,28^ Regulation of MMP activation is mediated via many different mechanisms including regulation of their tissue inhibitors, and their gene expressions by transcriptional factors and epigenetic modifications. Both, histone and DNA modifications in the *MMP-9* promoter region, initiated by the diabetic milieu, are shown to regulate its activation in the retina.^11,20^ Here, we report that there is a crosstalk between histone modification and DNA methylation in regulating *MMP-9* transcription. While the levels of histone repressor mark H3K27me3 are elevated and the enzyme methylating H3K27, Ezh2, is activated, due to increased recruitment of Dnmt1 by Ezh2 at the transcriptional factor binding site, the dynamic DNA methylation of *MMP-9* promoter is initiated. The levels of 5hmC are increased at the promoter, and this ultimately results in the transcriptional activation of *MMP-9*.

Histone methylation and DNA methylation are considered to be tightly coordinated; methylation of lysine can initiate, target, or maintain DNA methylation, and this is true vice versa as well.^29,30^ Our recent work has shown that the methylation status of *MMP-9* promoter is altered in diabetes, despite increased recruitment of Dnmt1 at the *MMP-9* promoter, 5mC levels are decreased. However, due to simultaneous activation of the hydroxymethlating Tet2 of the Tet family, the *MMP-9* promoter remains hypomethylated, suggesting an active cytosine methylation-demethylation process.^20^ Ezh2 can enhance DNA methylation by recruiting Dnmt1 at the promoter of a gene^18^; and the results presented here suggest a clear crosstalk between Ezh2 and DNA methylation. We show that the hyperglycemic environment favors binding of Ezh2 at the *MMP-9* promoter, which facilitates the recruitments of Dnmt1 and Tet2, ultimately, leaving the promoter hydroxymethylated, and activating *MMP-9* transcription. In support, recruitment of Dnmt1 by Ezh2 at the *ABCA1* promoter is shown to transcriptionally silence ABCA1 expression, and accelerate progression of atherosclerosis.^31^

Unlike acetylation, which is generally associated with gene activation, methylation of histones can either activate or inhibit gene transcription depending upon the site of methylation, and the degree of methylation.^32^ Among different histone modification scenarios, modifications of lysine 4, 9, and 27 of histone 3 are considered to be the most important histone modifications in influencing gene expression.^33,34^ In diabetes, H3K9me2 levels are decreased at the retinal *MMP-9* promoter and acetylated H3K9 is increased.^11^ Here, we show that, H3K27me3 levels are also significantly elevated, suggesting that multiple lysines at the same histone 3 in the *MMP-9* promoter are being affected in diabetes. Consistent with this, in smooth muscle cells, methylation status of all three lysine residues (H3K4, H3K9, and H3K27) are significantly altered in atherosclerosis; while methylation of H3K9 and H3K27 is decreased, that of H3K4 is increased.^35^

The enzyme responsible for trimethylation of H3K27, Ezh2, also serves as a catalytic subunit of PRC2.^18^ Our results demonstrate that diabetes increases H3K27me3 and activates Ezh2. In support, others have shown increased *Ezh2* mRNA in HRECs exposed to high glucose suggesting its role in regulation of VEGF.^17^ However, high glucose–exposed human podocytes have lower Ezh2,^36^ and in diabetic rats, despite increase in kidney Ezh2, H3K7me levels are not altered, suggesting an alternate mechanism.^37^ In addition to activation of Ezh2 in the retinal capillaries in hyperglycemic milieu, its recruitment at the *MMP-9* promoter is also increased. Inhibition of Ezh2 ameliorated its glucose-induced recruitment at the *MMP-9* promoter and decreased MMP-9 mRNA and activity; the reason for decreased Ezh2 binding at the *MMP-9* promoter by DZNep could be its degradation by DZNep, reducing Ezh2 protein levels.^38^ Our previous work using *MMP-9* knock-out mice has clearly shown that in diabetes, in addition to the retina being protected from accelerated capillary cell apoptosis and pathology characteristic of retinopathy, they have normal mitochondrial structure and mtDNA transcription. The same phenomenon is also observed in the retinal endothelial cells manipulated for MMP-9 activation.^6,7^ Consistent with this, Ezh2 inhibition also attenuates glucose-induced increase in mitochondrial localization of *MMP-9*, and protects mtDNA transcription, further confirming the role of Ezh2-H3K27me3 in regulation of *MMP-9-*mitochondrial damage. In support, regulation of *MMP-9* expression by Ezh2 in endothelial cells is implicated in the maintenance of the integrity of the developing vasculature.^16^

In the pathogenesis of diabetic retinopathy, *MMP-9* promoter, in addition to histone modifications, also undergoes DNA methylation-hydroxymethylation.^11,20,39^ Although DNA methylation and histone modification are mediated by different sets of enzymes, these epigenetic modifications appear to be biologically interrelated, and the relationship can work in either direction. For example, histone methylation can direct DNA methylation patterns, and DNA methylation can serve as a template for some histone modifications after DNA replication.^40^ H3K27me3 itself is a repressive mark associated with gene repression,^41^ but Ezh2 can also control DNA methylation directly by regulating Dnmts.^42,43^ Here, we show that despite increased H3K27me3 at the *MMP-9* promoter, its transcription is increased in diabetes. Regulation of Ezh2, along with inhibiting the binding of Dnmt1, also attenuates concomitant recruitment of Tet2 and 5hmC levels, which ultimately results in repression of *MMP-9* transcription. In support, overexpression of Ezh2 is shown to increase recruitment of Dnmts at the *TIMP2* promoter in ovarian cancer.^44^

Consistent with the results obtained from retinal endothelial cells in culture, retinal microvessels from diabetic rats also have increased *MMP-9* transcription,^7,11^ and we show that *MMP-9* promoter has increased H3K27me3 and Ezh2 recruitment, confirming similar phenomenon in animal model of diabetic retinopathy. In addition, transitioning to the human disease, our exciting results show that similar increase in H3K27me3 and Ezh2 is also observed in the retinal microvessels from human donors with diabetic retinopathy. This is accompanied by increased recruitment of both Dnmt1 and Tet2, and elevated levels of 5hmC, keeping *MMP-9* transcriptionally active. These results further confirm the role of Ezh2-H3K27me3-DNA methylation in the development of diabetic retinopathy.

Our study was focused on crosstalk between Ezh2-H3K27me3 and DNA methylation-demethylation of the *MMP-9* promoter, however, we acknowledge the role of demethylases in regulating H3K27me3 levels. In addition, similar crosstalk between histone methylation-DNA methylation of the intracellular inhibitor of MMP-9, TIMP1, in regulating MMP-9 activity also cannot be ruled out; Ezh2-mediated transcriptional repression of *TIMPs* is considered to be one of the major mechanisms shifting the MMPs-TIMPs balance and *MMPs* activation in invasive prostate cancer,^45^ and as mentioned above, in ovarian cancer, overexpression of Ezh2 increases recruitment of Dnmts at the *TIMP2* promoter.^44^

In summary, using experimental models of diabetic retinopathy, and confirming results in the retinal microvessels from human donors with diabetic retinopathy, this study provides strong evidence of a crosstalk between histone and DNA modifications in the regulation of *MMP-9* expression in diabetes. The results show that the recruitment of Ezh2 is increased at the *MMP-9* promoter in diabetes. This facilitates the recruitment of Dnmt1-Tet2, ultimately resulting in transcriptional activation of *MMP-9*, and regulation of Ezh2 protects DNA methylation, attenuating *MMP-9* transcription. Thus, in the pathogenies of diabetic retinopathy, MMP-9 has a major role in damaging the mitochondria and accelerating the apoptotic machinery,^6^ and Ezh2 appears to be critical in maintenance of cellular epigenetic integrity by regulating both histone modifications and DNA methylation.
